# Predictive models of glucose control: roles for glucose-sensing neurones

**DOI:** 10.1111/apha.12360

**Published:** 2014-08-28

**Authors:** C. Kosse, A. Gonzalez, D. Burdakov

**Affiliations:** Division of Neurophysiology, MRC National Institute for Medical Research, London, UK

**Keywords:** brain, glucose, hypothalamus

## Abstract

The brain can be viewed as a sophisticated control module for stabilizing blood glucose. A review of classical behavioural evidence indicates that central circuits add predictive (feedforward/anticipatory) control to the reactive (feedback/compensatory) control by peripheral organs. The brain/cephalic control is constructed and engaged, via associative learning, by sensory cues predicting energy intake or expenditure (e.g. sight, smell, taste, sound). This allows rapidly measurable sensory information (rather than slowly generated internal feedback signals, e.g. digested nutrients) to control food selection, glucose supply for fight-or-flight responses or preparedness for digestion/absorption. Predictive control is therefore useful for preventing large glucose fluctuations. We review emerging roles in predictive control of two classes of widely projecting hypothalamic neurones, orexin/hypocretin (ORX) and melanin-concentrating hormone (MCH) cells. Evidence is cited that ORX neurones (i) are activated by sensory cues (e.g. taste, sound), (ii) drive hepatic production, and muscle uptake, of glucose, via sympathetic nerves, (iii) stimulate wakefulness and exploration via global brain projections and (iv) are glucose-inhibited. MCH neurones are (i) glucose-excited, (ii) innervate learning and reward centres to promote synaptic plasticity, learning and memory and (iii) are critical for learning associations useful for predictive control (e.g. using taste to predict nutrient value of food). This evidence is unified into a model for predictive glucose control. During associative learning, inputs from some glucose-excited neurones may promote connections between the ‘fast’ senses and reward circuits, constructing neural shortcuts for efficient action selection. In turn, glucose-inhibited neurones may engage locomotion/exploration and coordinate the required fuel supply. Feedback inhibition of the latter neurones by glucose would ensure that glucose fluxes they stimulate (from liver, into muscle) are balanced. Estimating nutrient challenges from indirect sensory cues may become more difficult when the cues become complex and variable (e.g. like human foods today). Consequent errors of predictive glucose control may contribute to obesity and diabetes.

The brain can influence blood glucose levels independently of food intake and energy expenditure. This is illustrated, for example, by experiments where animals can be trained to lower their blood glucose in response to arbitrary cues (reviewed and discussed in Woods 2013). In this article, we discuss frameworks that may unify such classic behavioural data with the recently acquired knowledge of hypothalamic neural circuits involved in sensing and control of glucose levels.

The brain is thought to control blood glucose by sending signals (via the autonomic nerves) that make glucose shuttle between peripheral organs and the plasma. For example, the firing of sympathetic autonomic nerves can release glucose from liver stores into the blood, while parasympathetic nerves can stimulate the pancreas to produce insulin to lower blood glucose. A distinct property of this neural glucose control is that it is not just a reactive/corrective response to a change in glucose that already occurred. It is an anticipatory action based on an estimation of a future change in glucose level (Teff 2011, Woods 2013), that is a Pavlovian-like process (Pavlov 1902). Indeed, the lateral hypothalamus, which controls food choice and glucose levels as described below, also contains neurones that respond to food-associated cues (sight, taste) during hunger but not satiety, that is only when the animal is likely to ingest the food (Burton *et al.* 1976, Mora *et al.* 1976, Rolls *et al.* 1976).

Forward-planning actions of the brain (e.g. releasing insulin before food ingestion, Power & Schulkin 2008) would make glucose control more efficient, which can be viewed as an evolutionary advantage. Such actions are best known by terms ‘anticipatory’ or ‘cephalic’ in physiology, but are conceptually similar to feedforward/predictive controls in other evolvable systems (Franklin & Wolpert 2011, DiStefano *et al.* 2012). In this review, we will use ‘predictive’ to mean feedforward/anticipatory/cephalic. The general arrangement and usefulness of predictive and reactive controls is illustrated in Figure 1. A key advantage of reactive control (such as of glucose levels by the pancreas) is often described as disturbance resistance of controlled parameter (glucose level), which arises from self-correcting nature of feedback (Fig. 1a). A key disadvantage of feedback control is slowness, as it requires many steps: an event (eating), change in controlled parameter (glucose), sensing of this change by controller (pancreas) and reaction of controller (insulin release). Feedforward control has a complementary set of advantages and disadvantages: it is fast but blind to its output (Fig. 1b). A predictive controller triggers control actions before the event, usually using neutral ‘event cues’ (e.g. a sudden noise) to drive anticipatory actions, such as hepatic glucose release to fuel potential escape actions. Together, predictive and reactive strategies thus form a useful control scheme, both fast and self-correcting. This could be an evolutionary rationale for why the peripheral organs and the brain came to cooperate in controlling glucose levels (Fig. 1c).

In this review, we try to unify this general logic of control with specific experimental knowledge of brain neurocircuits regulating glucose levels. For good predictive control, the brain has to estimate how likely an event (or a neutral event cue) is to change blood glucose level. This requires associative learning, and the ability to refine and update it based on experience (in this case, experience of actual glucose changes). We will discuss a circuit model where predictive control, including associative learning, is linked to glucose-sensing neurones. As background for this model, we first review experimental data on brain glucose-sensing neurones. Our overall intention is to discuss a general systems view (von Bertalanffy 2013) of brain glucose control and glucose-sensing neurones. For more comprehensive account of cellular/molecular components and processes controlling energy balance, the reader is referred to other sources (e.g. Berthoud 2011, Yeo & Heisler 2012, Petrovich 2013). Although we do not discuss all known data, to the best of our knowledge, current experimental measurements do not contradict the hypotheses outlined here.

## Glucose-sensing neurones and physiological changes in extracellular glucose levels

**Figure 2:**
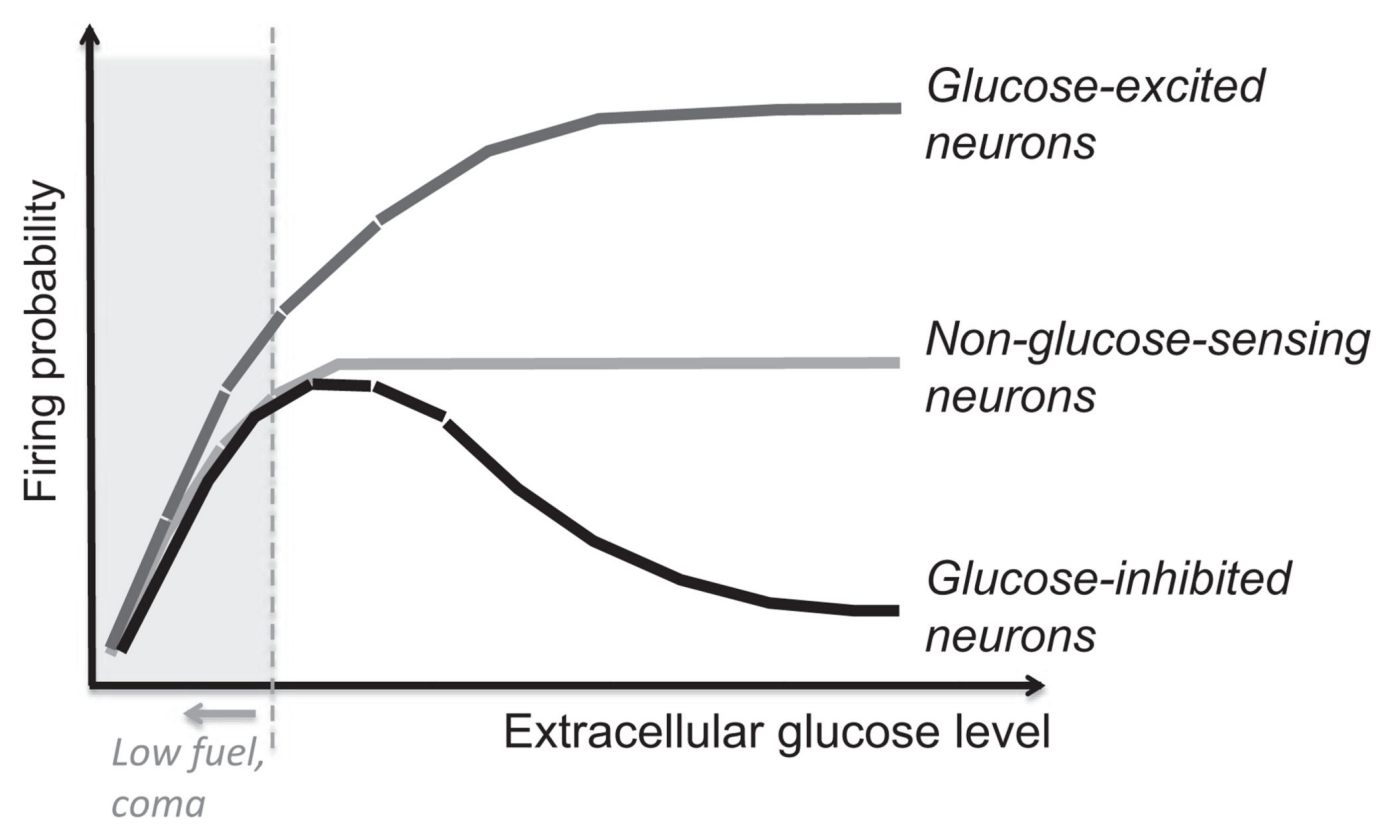
Graphical depiction of ubiquitous responses to low glucose vs. specialized glucose-sensing responses of neurones.

Discovery of glucose-sensing neurones in brain areas such as the hypothalamus, brain stem and substantia nigra suggested that the brain can directly monitor body energy status (Anand *et al.* 1964, Oomura *et al.* 1969, 1974, Yuan *et al.* 2004). The firing of ‘glucose-excited’ and ‘glucose-inhibited’ neurones is steeply tuned within ambient glucose range (e.g. 1–5 mm, Burdakov *et al.* 2005). This concentration range is above saturation of ubiquitous glucose transporters and hexokinases (Qutub & Hunt 2005). Glucose-sensing responses are thus distinct from ubiquitous silencing of neurones by low glucose (Fig. 2). They are not seen in most neurones in the brain, whose glucose-phosphorylating enzymes are thought to have km values of around 40 μm, and so presumably saturate and do not change intracellular energy levels upon glucose fluctuations >1 mm (Qutub & Hunt 2005).

Biophysical properties of glucose-sensing neurones, their similarities to peripheral glucose-sensing cells such as pancreatic insulin-secreting cells and the still unclear intracellular transduction mechanisms for glucose-sensing have been discussed in other recent reviews (Mobbs *et al.* 2001, Routh 2002, Karnani & Burdakov 2011). Here, we only briefly recapitulate the evidence that brain glucose levels change within a range sensed by glucose-sensing neurones. In rodents, simultaneous measurements of glucose in brain and blood show that brain glucose levels are generally 10–30% of blood glucose levels (Silver & Erecinska 1994, Routh 2002, Dunn-Meynell *et al.* 2009). However, in this lower range, brain glucose levels rapidly follow changes in blood glucose levels (Silver & Erecinska 1994). At euglycemia, brain glucose levels are thought to be around 0.7–2.5 mm (Routh 2002). A maximum of around 5 mm may be reached during severe plasma hyperglycaemia, while during hypoglycaemia, brain glucose may fall to 0.2–0.5 mm (Silver & Erecinska 1994). A point of continuing controversy is whether brain glucose levels approach blood glucose levels near areas of high permeability in the blood–brain barrier (circumventricular organs), such as the median eminence in the hypothalamus (Ganong 2000). It has recently been shown that this may not apply to neurones in close vicinity, such as the arcuate nucleus (ARC) in the case of median eminence (Dunn-Meynell *et al.* 2009), perhaps, because of tanycyte cell barriers between the ARC and median eminence (Mullier *et al.* 2010; Peruzzo *et al.* 2000).

Meals are thought to increase blood glucose and thus also brain glucose, but the extent to which this happens depends on macronutrient content (Jenkins *et al.* 1981). Generally, meals high in protein or fat elevate blood glucose less than carbohydrate-rich meals. Another source of blood glucose is glucose release from the liver, which is also under brain control as discussed below. Hepatic glucose release is regulated by insulin and glucagon as well as, but to a lesser extent, directly by the autonomic nerves (Puschel 2004). Finally, blood glucose is also influenced by glucose uptake into muscle and adipose tissues, which is increased by insulin and sympathetic activity (Nonogaki 2000).

Thus, current evidence suggests that glucose-sensing neurones may sense physiological changes in ambient glucose (and in turn, control glucose levels via feeding and autonomic nerves, see below). Recent examinations of neurochemical identities of glucose-sensing neurones revealed diverse cells groups. For example, in the hypothalamus, glucose-inhibited neurones are thought to comprise anatomically and functionally distinct subgroups, for example neurones containing orexin/hypocretin (ORX, in the lateral hypothalamus), NPY/AgrP (in the hypothalamic arcuate nucleus), and SF-1 (in the hypothalamic ventromedial nucleus). Glucose-excited neurones may include POMC neurones (in the arcuate nucleus) and melanin-concentratinghormone, (MCH, in the lateral hypothalamus) (Karnani & Burdakov 2011). Some brain areas outside the lateral and mediobasal hypothalamus have also been shown to contain glucose-sensing neurones (Ritter *et al.* 1981, Adachi *et al.* 1984, Melnick *et al.* 2011), but their identities are incompletely understood.

In this review, we will limit our discussion to MCH and orexin neurones of the lateral hypothalamus and attempt to present experimental knowledge about their functions within a broader context of predictive glucose control by the brain. We acknowledge that it is not yet clear whether other glucose-sensing neurones are conceptually similar to the roles of orexin and MCH cells hypothesized here and whether orexin and MCH neurones function as homogenous or heterogenous populations in relation to glucose control.

## MCH neurones and predictive control

Recent data suggest that MCH neurones are essential for learning to select nutritionally superior foods based on taste, a useful predictive operation (Domingos *et al.* 2013). Taste itself is a neutral cue; it has no nutrient value. However, it can be rapidly detected by tongue receptors, making it a useful trigger for prediction-based actions (such as food choice), after learning that eating foods with certain tastes produces nutrients. The animal may thus learn a more time-efficient food selection strategy, by developing a ‘cognitive shortcut’ directly from taste to food selection. The basic idea suggested by recent experiments (reviewed below) is that activation of MCH neurones – possibly in part by glucose – is an essential permissive signal for building such a shortcut inside the brain. We first review basic properties of MCH neurones relevant to this role. Then, we put them into the context of using nutrient value of sugar to construct predictive control circuits for ingestive behaviour.

### Neurochemical, anatomical and behavioural overview of MCH neurones

MCH-expressing neurones are located exclusively in the lateral hypothalamus, but project widely throughout the brain (Bittencourt 2011). At their projection targets, MCH neurones are thought to release inhibitory transmitters. These transmitters include MCH-related peptides (which act on specific G-protein-coupled receptors), as well as GABA, whose release during MCH cell firing can affect target neurones very fast (milliseconds), by activating GABA-A receptors (Jego *et al.* 2013).

Like ORX, MCH has been often viewed as a feeding–promoting transmitter. However, in many ways, actions of MCH cells appear the opposite to those of ORX cells. For example, knockout of MCH in mice increases energy expenditure and reduces body weight (Shimada *et al.* 1998), which is recapitulated by knockout of the MCH receptor MCH1R, the only known MCH receptor in mice (Marsh *et al.* 2002). MCH neurones are thought to fire mostly during REM sleep, whereas ORX neurones are thought to fire mostly during active waking (Hassani *et al.* 2009, 2010). Brain injection of MCH promotes sleep, as does optogenetic stimulation of MCH neurones (Verret *et al.* 2003, Jego *et al.* 2013, Konadhode *et al.* 2013), while deletion of MCH or MCH1R leads to increased locomotor activity (Zhou *et al.* 2005). Thus, endogenous MCH may promote sleep and suppresses locomotor activity and energy expenditure, that is the opposite of actions of ORX. While MCH neurones are often called ‘appetite promoting’, their role in food intake is not completely obvious. Knockout of MCH in (nocturnal) mice increases food intake during the day, but not during the night (Shimada *et al.* 1998). Not all (Presse *et al.* 1996) studies show that MCH increases food intake, and mice lacking MCHR1 actually have *increased* food intake (Chen *et al.* 2002, Marsh *et al.* 2002).

There is a growing realization that glucose homeostasis and metabolic health depend on sleep patterns (Bass & Takahashi 2010, Reddy & O’Neill 2010). Good predictive control requires learning, which is also closely linked to sleep (Diekelmann & Born 2010). In this context, it is interesting that the firing of MCH neurones has been recently causally liked to sleep (Jego *et al.* 2013, Konadhode *et al.* 2013). Activation of MCH neurones could prolong REM sleep episodes, while silencing MCH neurones led to termination of REM (Jego *et al.* 2013). This raises an intriguing possibility that electrical activity of MCH cells is necessary and sufficient for REM sleep. Release of both GABA and MCH peptides from MCH cells, on targets such as wake-promoting hypothalamic histamine neurones as well as hippocampal circuits, is probably involved in this sleep control (Jego *et al.* 2013). Interestingly, MCH also increases the beat frequency of cilia lining a part of the third ventricle (Conductier *et al.* 2013), suggesting that it may sustain CSF movement and circulation during REM sleep, when body movement is minimal due to REM-associated muscle atonia.

### Glucose sensing and control by MCH neurones

Physiologically relevant increases in local extracellular glucose levels have been shown to excite MCH neurones in mouse brain slices (Burdakov *et al.* 2005, Kong *et al.* 2010). More recently, this excitation has been shown to involve glucose-dependent closure of Kir6.2/SUR1-containing ATP-inhibited K^+^ (K-ATP) channels (Kong *et al.* 2010). Expression of ATP-resistant K-ATP channels selectively in MCH neurones ablated MCH cell glucosensing and impaired glucose tolerance (Kong *et al.* 2010). While this suggests that glucose excitation of MCH neurones may improve glucose tolerance, the acute v chronic role of MCH neurones in glucose control is unclear at present. For example, although glucose tolerance worsens if MCH cells are made glucose-insensitive from birth (Kong *et al.* 2010), it actually improves if MCH cells are destroyed in the adult (Whiddon & Palmiter 2013). Furthermore, MCH overexpression leads to obesity and insulin resistance (Ludwig *et al.* 2001) and increases insulin levels (Pissios *et al.* 2007). It is possible that, like ORX neurones, MCH cells could have multiple, differentially modulated effects on glucose release and uptake. Alternatively, as discussed below, the actions of MCH neurones could be more central for coordinating predictive glucose control, rather than for reactive responses to increased glucose levels.

### MCH neurones, learning and memory

Learning, especially formation of associative memories, is a prerequisite for good predictive control. Among projection targets of MCH neurones are several memory-related structures, including the hippocampus and septum (Adamantidis & de Lecea 2009, Jego *et al.* 2013), where activation of MCH receptors may promote synaptic strengthening (Pachoud *et al.* 2010). MCH neurones directly project to the hippocampal formation and may regulate hippocampal function through the medial septum nucleus. Sleep is thought to be important for memory, and release of acetylcholine in the hippocampus is considered vital for memory consolidation. Central administration of MCH peptide has been shown to prolong REM and non-REM sleep episodes and to increase hippocampal, but not cortical, acetylcholine release (Lu *et al.* 2013).

### MCH neurones and associative learning for predictive control

As outlined above, good predictive control requires learning an association between a neutral but easily measurable parameter (‘conditioned stimulus’ in psychology) and actual nutrient value of food (‘unconditioned stimulus’, cannot be measured quickly for most food). Subsequently, detection of a conditioned stimulus can be used for efficient (i.e. rapid) action selection and for preparing the body for subsequent slower arrival of glucose to blood. Recent experimental evidence suggests that MCH neurones are a key part of such associative learning.

For example, Sherwood *et al.* found that genetic or pharmacological disruption of the MCH receptor (MCH-1R) reduced the ability of a previously learnt conditioned stimulus to serve as a reinforcer in a new instrumental behaviour task (Sherwood *et al.* 2012). This raises the possibility that MCH signalling may be important for the response transfer in dopamine neurones (Schultz *et al.* 1997), which shift their activity from occurrence of the reward to the earliest conditioned stimulus, while the stimuli closer to the reward (e.g. taste) serve as conditioned reinforcers for earlier occurring stimuli (e.g. colour, shape of food).

More recently, Domingos *et al.* (2013) obtained data suggesting that MCH neurones are necessary for a conditioned stimulus, taste, to evoke brain reward signals (and thus presumably action selection). In these experiments, mice naturally preferred calorie-containing sucrose to non-caloric sweetener sucralose. This is thought to be because metabolisable sugar enabled sweet taste to generating a brain reward signal, dopamine (Domingos *et al.* 2013). Optogenetic stimulation of MCH neurones effectively substituted for metabolizable sugar, so mice came to prefer sucralose when its consumption was paired with MCH neurone activation (Domingos *et al.* 2013). This suggests that MCH neurone activation [presumably at least in part by glucose as tasting artificial sweeteners does not activate MCH neurones (Shiuchi *et al.* 2009)] instructs reward learning when sweet taste leads to nutrients. Loss of MCH neurones stopped mice from preferring sucrose to sucralose. These data are consistent with conclusions of an earlier pharmacological study showing that blocking the MCH receptor reduces lever pressing reinforced by sucrose, but not lever pressing reinforced by artificial sweetener (Karlsson *et al.* 2012).

In mice with normal sweet taste receptors, activation of MCH neurones did not drive reward unless sweet taste receptors were also stimulated (Domingos *et al.* 2013), suggesting that in normal mice, both MCH neurones and taste are required for establishing a behavioural preference. This implies the existence of conditional switch circuits (also called AND logic gates) that either allow MCH neurones to be activated by sugar only when taste input is present, or allow dopamine release only when both MCH input and taste input are present. Currently, the latter seems more likely, because in normal mice, MCH neurone activation did not drive DA release and behavioural preference without taste (Domingos *et al.* 2013). However, there are probably alternative pathways (not involving taste signalling) for enabling MCH neurones to facilitate food preference, as ‘sweet-blind’ Trpm5-/- mice learn to prefer sucrose to sucralose in a manner dependent on MCH neurones (Domingos *et al.* 2013).

**Figure 3:**
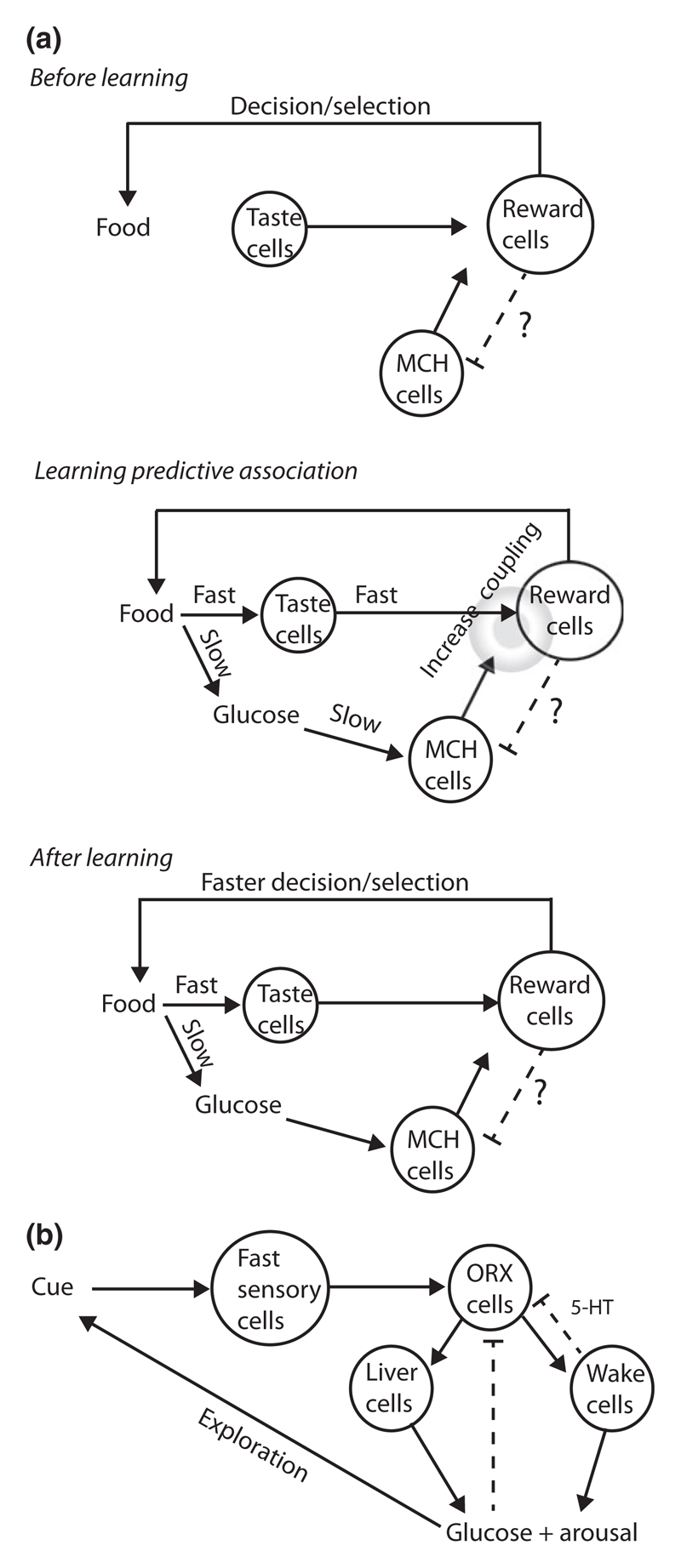
Cell-based models for orchestrating predictive glucose control by melanin-concentrating hormone (a) and orexin (ORX) (b) neurones. In a, dashed line with question mark shows hypothetical negative signal from reward cells (unknown, not discussed here) that might be required for controlling learning based on existing and future associations (Schultz & Dickinson 2000). In b, Cue-ORX coupling might also be subject to learning via unknown pathways not discussed here. Arrows depict excitation, and t-bars depict inhibition.

Overall, current evidence therefore suggests that, by controlling synaptic plasticity, MCH neurones may gate the ability of neutral cues (e.g. taste) to elicit brain reward and action selection, presumably based on associated changes in energy levels (Fig. 3a).

## ORX neurones and predictive control

ORX neurones are activated by diverse stimuli that are of no nutrient value themselves, but may indicate that energy intake or expenditure is imminent [e.g. sounds, sweet taste, stress (Mileykovskiy *et al.* 2005, Shiuchi *et al.* 2009, Winsky-Sommerer *et al.* 2004)]. This fits the operational definition of predictive control action, considering that ORX neurones stimulate many ‘fight-or-flight’ physiological responses [e.g. arousal, sympathetic drive, heart rate, flux of glucose from liver to muscles (Karnani & Burdakov 2011, Kuwaki 2011, Grimaldi *et al.* 2014)]. Below, we review experimental investigations of ORX cell properties, from a viewpoint that ORX neurones are part of predictive trigger circuits for adaptive physiological responses.

### Neurochemical, anatomical and behavioural attributes of ORX neurones

ORX neurones have been subject of detailed recent reviews (de Lecea *et al.* 2006, Sakurai 2007) and are introduced only briefly here. Like MCH neurones, in the brain, ORX neurones are located exclusively in the lateral hypothalamus, from which they innervate a long list of cortical and subcortical brain areas (Peyron *et al.* 1998), including the cerebellum (Nisimaru *et al.* 2013). A net impact of ORX neurone on physiological set points can be summarized as keeping mammals ‘thin and awake’, as loss of ORX neurones produces narcolepsy and obesity (Hara *et al.* 2001, Dauvilliers *et al.* 2007). ORX neurones are not necessary for wakefulness *per se*, but are essential for stabilizing wakefulness into sustained episodes required for many behaviours. Thus, animals deficient in ORX neurones (or ORX type 2 receptors) still display relatively normal amounts of total sleep and total wake time; however, their wakefulness (and sleep) are fragmented (Hara *et al.* 2001). ORX neurones are therefore considered to be essential for adjusting arousal to the environment, as ORX-deficient narcoleptic animals (and humans) experience irresistible attacks of sleep. Causes of ORX cell loss in human narcolepsy are still debated, but increasingly thought to be at least partly autoimmune (De la Herran-Arita *et al.* 2013, Mahlios *et al.* 2013).

ORX neurones exert their effects on downstream circuits via two specific G-protein-coupled receptors for ORX peptide neurotransmitters (Sakurai 2007), as well as via release of glutamate that alters target neurone firing on a millisecond timescale (Schöne *et al.* 2012). The type 2 ORX receptor is particularly critical for stable wakefulness (Lin *et al.* 1999, Willie *et al.* 2003), whereas type 1 ORX receptor has been proposed to regulate reward and addiction (Boutrel *et al.* 2005, Cason & Aston-Jones 2013a,b, Mahler *et al.* 2013). The role of ORX neurones in driving reward seeking is well established, but many details (e.g. whether there are segregated ‘arousal’ and ‘reward’ ORX circuits) remain unclear and are under active investigation (Harris & Aston-Jones 2006, de Lecea *et al.* 2006, Gonzalez *et al.* 2012). It is generally agreed, however, that ORX neurones can engage arousal and reward-seeking drive together, leading to exploratory behaviour. At the same time, ORX neurones stimulate breathing, circulation, glucose release from liver and glucose uptake by muscle, presumably to orchestrate energy supply for exploratory or escape behaviour (reviewed in detail in Grimaldi *et al.* 2014, Karnani & Burdakov 2011). Data about the activity profile of ORX neurones *in vivo* across behavioural states are sparse, but are consistent with the latter role of ORX cells, with higher activity during active waking, lower activity during quiet waking and little activity during sleep (Lee *et al.* 2005, Mileykovskiy *et al.* 2005).

### Activation of ORX neurones by cues predicting imminent glucose change

The firing of ORX neurones stimulates awakening (Adamantidis *et al.* 2007). However, during wakefulness, ORX neurones are not uniformly active and often show periods of complete silence (Mileykovskiy *et al.* 2005). Their firing is thus not essential for wakefulness *per se*, but is thought to boost wakefulness when demanded by the environment. ORX neurones are activated by diverse stimuli that may demand increased arousal, energy expenditure and goal-oriented behaviour, for example sudden sounds, tasting of artificial sweeteners, stress, hypoglycaemia and hypercapnia (Yamanaka *et al.* 2003, Winsky-Sommerer *et al.* 2004, Mileykovskiy *et al.* 2005, Williams *et al.* 2007, Shiuchi *et al.* 2009). A common feature of these stimuli is that they predict a change in glucose consumption, which is likely to be needed to overcome an imminent challenge. This suggests that ORX neurones are an essential part of a predictive control circuit that primes the brain and the body for action, even in response to neutral stimuli such as sounds. Indeed, when actions of ORX neurones are experimentally suppressed, key predictive adaptations to the environment are reduced, such as blood pressure rises in resident-intruder tests’ and fasting-induced exploration (Kayaba *et al.* 2003, Yamanaka *et al.* 2003).

### Inhibition of ORX neurones by glucoset

Measurements of genetic markers of neuronal activation and of ORX mRNA levels following *in vivo* manipulation of glucose levels suggest that ORX neurones are activated by hypoglycaemia and are thus ‘glucose-inhibited’, considering they do not directly respond to insulin (Moriguchi *et al.* 1999, Cai *et al.* 2001, Yamanaka *et al.* 2003). Initially, it was found that orexin-A/hypocretin-1 was not present in glucose-inhibited neurones of the lateral hypothalamus (Liu *et al.* 2001), and a more recent electrophysiological study also failed to observe glucose responses in rat ORX neurones (Parsons & Hirasawa 2010). However, at least three different groups reported robust acute inhibition of ORX neurones by glucose, using calcium imaging of rat ORX cells, or patch-clamp recordings from mouse ORX cells (Muroya *et al.* 2001, Yamanaka *et al.* 2003, Williams *et al.* 2008, Guyon *et al.* 2009).

The discrepancies between different groups’ data on glucose-sensing capability of ORX neurones may be related to two experimentally documented features of this sensing response: adaptation and context dependence. *In vitro*, some ORX cells can adaptively reduce their glucose-stimulated K^+^ conductance when glucose levels are stable. After an inhibition caused by a glucose rise, they intrinsically reset to their prior excitability level even though glucose is still ‘up’ (Williams *et al.* 2008). Such stimulus adaptations are common across sensory systems (Carpenter 2003) and may help ORX cells to maintain glucose-trend sensing irrespective of basal glucose level (Williams *et al.* 2008). The context dependence of ORX cell glucose sensing is illustrated by *in vitro* findings that their ability to sense glucose dose dependently disappears upon increased levels of lactate, pyruvate or ATP (Venner *et al.* 2011), upon reduced extracellular pH (Burdakov *et al.* 2006) or (through pH-independent effects) by the presence of nutritionally relevant mixes of dietary amino acids (Karnani *et al.* 2011). Thus, under some circumstances, ORX cells may turn off their glucose-sensing ability in favour of integrating other signals.

Overall, current evidence therefore suggests that ORX neurones are part of a predictive–reactive circuit, which can output arousal and energy for potential actions based on neutral cues, but is also subject to feedback inhibition by some of these outputs (Fig. 3b).

## Unifying model for glucose-sensing neurones and predictive–reactive glucose control

**Figure 4:**
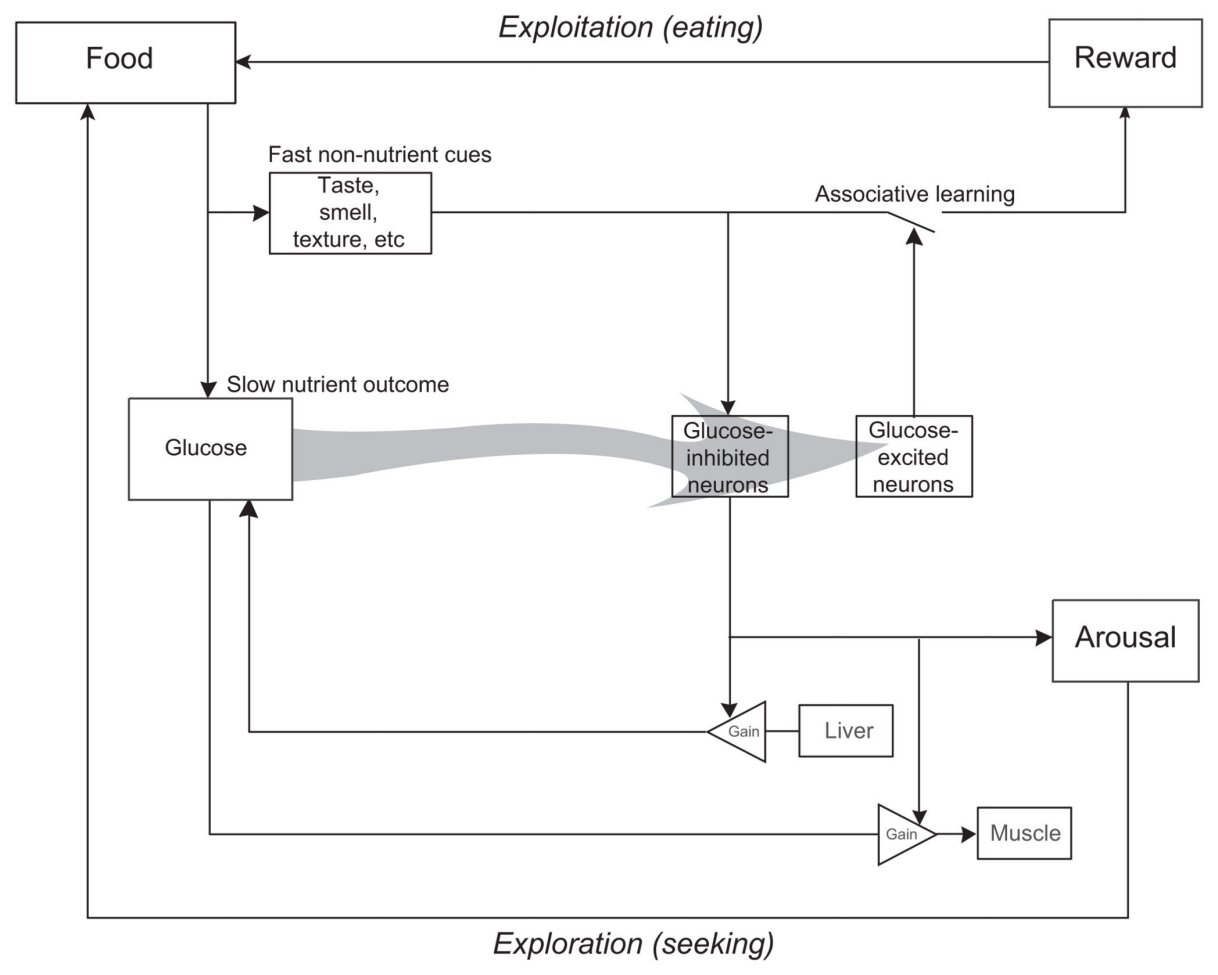
Block diagram model for orchestration of central and peripheral processes by glucose-sensing neurones, in the context of reactive and predictive control of glucose levels. Glucose-inhibited neurones, such as orexin cells, may facilitate exploration identify prospective nutrient sources. Glucose-excited neurones, such as melanin-concentrating hormone cells, may facilitate exploitation of prior knowledge for efficient food selection based on ‘fast’ (readily measurable) cues. Glucose level may thus shift the balance between exploration and exploitation.

The reviewed evidence suggests that glucose-excited and glucose-inhibited neurones are an integral part of predictive glucose control by the brain (Fig. 4). More specifically, we hypothesize that during associative learning, inputs from some glucose-excited neurones may promote connections between the ‘fast’ senses and brain reward circuits. This would enable reward signals to be generated in response to cue rather than to later outcome (Schoenbaum *et al.* 2013), thereby constructing neural shortcuts for efficient action selection. Apart from glucose-excited MCH neurones reviewed above, other glucose-excited neurones may also contribute to this. For example, glucose-excited POMC neurones of the hypothalamic arcuate nucleus innervate memory and reward centres (O’Donohue *et al.* 1979), and peptides they release are implicated in synaptic plasticity (Caruso *et al.* 2014).

In turn, glucose-inhibited neurones may engage locomotion/exploration and coordinate the required fuel supply. Feedback inhibition of the latter neurones by glucose would ensure that glucose fluxes they stimulate (from liver, into muscle) are balanced (Fig. 4). Apart from glucose-inhibited ORX neurones reviewed above, other putative glucose-inhibited neurones may also play this role, for example SF-1 neurone of the ventromedial hypothalamus (Karnani & Burdakov 2011).

## Questions, limitations and implications

Our model unifies diverse experimental data with a control framework for improving glucose control. It is hoped that this may produce a clearer view of outstanding questions and discrepancies, and help to generate testable hypotheses. Many unknowns remain. For example, currently available data suggest that MCH cell firing, which enables taste to drive reward (Domingos *et al.* 2013), occurs only during sleep, especially REM sleep (Hassani *et al.* 2009). Does this mean that associative learning for predictive glucose control occurs only during sleep? Current data also suggest that MCH cells help to construct predictive control circuit in the brain (Domingos *et al.* 2013), but in contrast to ORX neurones, MCH cells are not themselves activated by neutral cues such as taste (Shiuchi *et al.* 2009, Petrovich *et al.* 2012). It is also not clear whether learning plays a role for the predictive adaptations initiated by ORX neurones, but it might be possible as orexin/ataxin-3-transgenic mice show impaired long-term potentiation in hippocampus (Yang *et al.* 2013); and in the VTA, drug-induced plasticity of glutamate synapses onto dopamine neurones can be promoted by orexin (Baimel *et al.* 2014). Do ORX and MCH neurones operate simultaneously and in parallel or are their actions segregated in time, as suggested by their reciprocal firing profiles during sleep and wakefulness (Hassani *et al.* 2009), and the finding that MCH neurones are inhibited by direct projections from histaminergic neurones in wake-promoting regions (Parks *et al.* 2014)?

We did not explicitly discuss circuitry for updating associations involving MCH neurones [e.g. via prediction error-based learning, (Schultz & Dickinson 2000)], or the role of learning in predictive circuit involving ORX neurones. Little experimental data currently exist on these vital aspects of hypothalamic control systems, but additional cells and processes are likely to be required to account for this (Schultz & Dickinson 2000). Delineating these circuits would be important for understanding how predictive beliefs about food can be reshaped. We also note that, although it is clear that ORX and MCH neurones are involved in predictive control, little data yet exist (with the exception of Kong *et al.* 2010) quantifying the impact of their direct glucose responses on glucose control efficiency.

Viewing central regulation of glucose as predictive control has implications for identifying origins of metabolic disease. Predictive control works best when associations between neutral stimuli (e.g. colour of food) and caloric value are consistent and so can be readily learnt and used for accurate predictive actions (e.g. releasing X amount of insulin upon seeing a red apple). Many of these associations were presumably quite stable on an evolutionary timescale. They are no longer fixed in the modern environment filled with diverse and rapidly changing food packaging and advertising (e.g. calorically identical food can come in many different packages), and artificial flavours mimicking taste without providing caloric value. Presumably, this poses a problem for predictive control, because estimating nutrient challenges from indirect sensory cues becomes different in this complex environment. Frequent errors of predictive control can presumably put a strain on reactive controllers (liver and pancreas) that correct these errors. We hypothesize that predictive control errors may contribute to the development of obesity and diabetes.

## Figures and Tables

**Figure 1:**
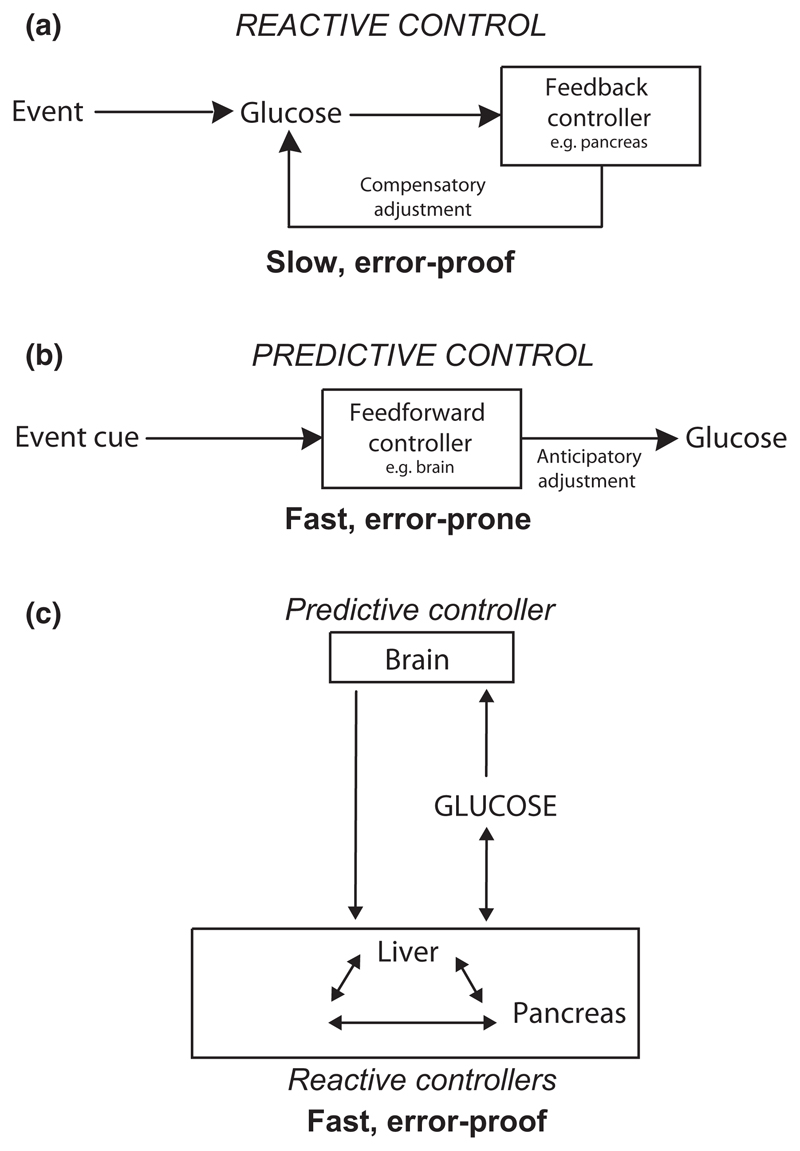
Canonical schemes for reactive (a) and predictive (b) adjustments in the context of glucose level control. Combining reactive and predictive control (c) overcomes limitations of individual control modes.
